# Crystal structure of 4-[(4-methyl­benz­yl)­oxy]-*N*′-(4-nitro­benzyl­idene)benzohydrazide: a new hydrazone derivative

**DOI:** 10.1107/S2056989023003948

**Published:** 2023-05-05

**Authors:** Md. Hasan Al Banna, Md. Chanmiya Sheikh, Ryuta Miyatake, Md. Belayet Hossain Howlader, Ennio Zangrando

**Affiliations:** a Department of Chemistry, Rajshahi University, Rajshahi-6205, Bangladesh; b Department of Applied Science, Faculty of Science, Okayama University of Science, Japan; c Center for Environmental Conservation and Research Safety, University of Toyama, 3190 Gofuku, Toyama, 930-8555, Japan; d Department of Chemical and Pharmaceutical Sciences, University of Trieste, Italy; Vienna University of Technology, Austria

**Keywords:** crystal structure, hydrazine, hydrazone

## Abstract

The mol­ecular structure of the title compound, C_22_H_19_N_3_O_4_, shows a non-coplanar conformation, with dihedral angles between the phenyl rings of 73.3 (1) and 80.9 (1)°. These deformations are induced by the crystal packing that is mainly governed by N—H⋯O and C—H⋯O hydrogen bonds, forming a mono-periodic arrangement parallel to the *b* axis.

## Abstract 2

The title aroylhydrazone ether exists in an *E*-configuration with respect to the double bond of the hydrazone bridge and with an ac­yl–hydrazone (—CH=N—NH—CO—) torsion angle of 166.0 (3)°. The mol­ecule exhibits a non-planar conformation, likely induced by packing requirements.

## 1. Chemical context

Hydrazones are a special class of Schiff bases, which can be obtained by condensation between an alkyl or aryl hydrazine and a carbonyl compound (aldehyde or ketone). The active pharmacophore group, —CH=N—NH—C=O—, present in a hydrazone is primarily responsible for its broad spectrum of biological aspects (Taha *et al.*, 2013 ▸). The presence of tautomeric forms facilitates their coordination behavior in neutral or anionic species (Banna *et al.*, 2022 ▸) with metal ions (Zülfikaroğlu *et al.*, 2020 ▸). The chemical diversity and pharmacological accessibility of hydrazone and its derivatives paves the way for research exploring drug design and discovery (Verma *et al.*, 2014 ▸). 
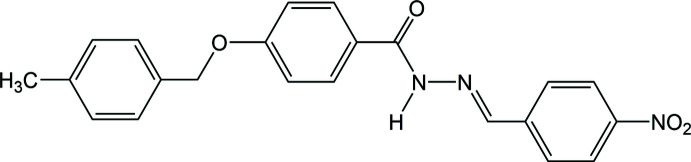


In this context and in a continuation of our recent work (Banna *et al.*, 2023 ▸), we report here on the synthesis and crystal-structure determination of another derivatized aroylhydrazone bearing an ether group.

## 2. Structural commentary

**Figure 1:**
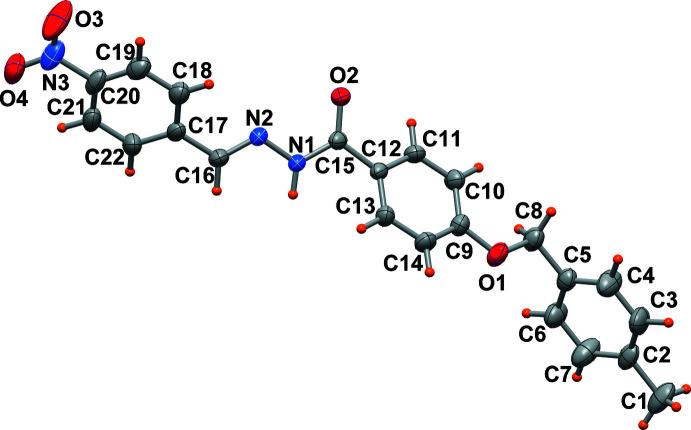
The mol­ecular structure of the title compound with displacement ellipsoids drawn at the 50% probability level.

The mol­ecular structure of the hydrazone compound is shown in Fig. 1 ▸. The ac­yl–hydrazone (—CH=N—NH—C=O—) group connects the *p*-nitro­phenyl group and the central phenyl ring, which in turn is bound to the *p*-methyl­benz­yloxy fragment. An *E*-configuration is observed with respect to the double bond of the hydrazone bridge N2=C16. The N1—N2 bond length of 1.376 (4) Å is slightly shorter than that of 1.397 (4) Å determined in the corresponding derivative having a thienyl ring replacing the *p*-nitro­phenyl group (Banna *et al.*, 2023 ▸). On the other hand, the O2=C15 bond of 1.237 (4) Å is close to that determined in the thienyl derivative [1.236 (4) Å], and typical of a ketonic linkage, while an equilibrium between the keto and enol forms is present in solution. The nitro­phenyl group and the benzohydrazone fragment form a dihedral angle of 73.3 (1)° while the terminal 4-methyl­benzyl group is rotated by 80.9 (1)° with respect to the central phenyl ring.

**Figure 2:**
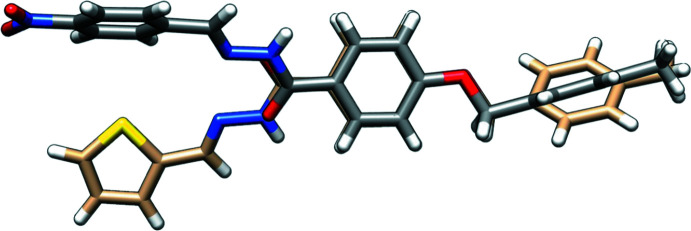
Overlay plot of the mol­ecule of the title compound and the reported thienyl derivative (Banna *et al.*, 2023 ▸).

Fig. 2 ▸ depicts a superimposition of the mol­ecular structure of the title compound with the thienyl derivative (Banna *et al.*, 2023 ▸). It is worth noting the different orientations of the carbohydrazide CO—NH—N moiety, likely induced by crystal-packing requirements.

## 3. Supra­molecular features

**Figure 3:**
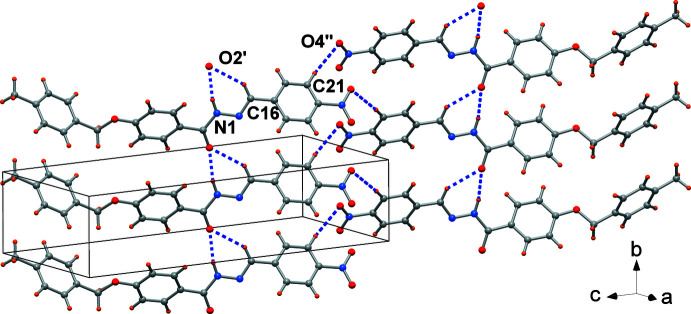
Crystal packing of the title compound showing the mono-periodic arrangement parallel to the *b* axis built by N—H⋯O and C—H⋯O hydrogen bonds (dashed lines).

The crystal packing is governed by hydrogen-bonding inter­actions (Table 1 ▸, with corresponding symmetry codes) realized between the imino group N1—H1 with carbonyl oxygen atom O2^ii^ of a symmetry-related mol­ecule. This results in a mono-periodic arrangement parallel to the *b* axis. In addition, non-classical C16—H16⋯O2^ii^ hydrogen bonds between a methine group and the carbonyl O atom and C21—H21⋯O4^iii^ between an aromatic C—H group and one of the nitro O atoms are also present, as shown in Fig. 3 ▸. The ribbons are further connected by C14—H14⋯N2^i^ inter­actions (Table 1 ▸). No significant π-stacking inter­action is found in the crystal (all centroid-to-centroid distances between phenyl rings are > 5.0 Å).

## 4. Database survey

For a closely related structure with a thienyl moiety, see: Banna *et al.* (2023 ▸); for some other aroylhydrazones, see: Ban & Li (2009 ▸); Chantrapromma *et al.* (2016 ▸); Horkaew *et al.* (2011 ▸); Zong & Wu (2013 ▸). All these mol­ecules exhibit an *E*-configuration about the double bond of the hydrazone bridge, and they have comparable bond lengths and angles in the C=N—NH—C moiety, in agreement with the present geometrical parameters. For reference bond-length data, see: Allen *et al.* (1987 ▸).

## 5. Synthesis and crystallization

The synthesis of the compound follows a procedure previously described (Banna *et al.*, 2023 ▸). To a solution of 4-[(4-methyl­benz­yl)­oxy]benzoyl­hydrazine (0.25 g, 0.97 mmol in 20 ml of absolute ethanol), a solution of 4-nitro­benzaldehyde (0.14 g, 0.97 mmol) in 5 ml ethanol was added and the mixture was heated and refluxed for 2 h. A colorless precipitate was obtained, filtered off, and washed several times with hot ethanol to eliminate any types of starting materials prior to being dried in a desiccator. The title compound was recrystallized from a mixture of DMF and ethanol. Colorless crystals suitable for X-ray diffraction were obtained after 60 d of keeping the sample solution undisturbed.

Yield: 0.29 g, 79%; melting point (m.p.): 531–533 K; FT–IR: 1636 ν(C=O_amide_), 3315 ν(N—H), 1606 ν(C=N_azomethine_). LC–MS (FAB) *m*/*z*: [*M* + H]^+^ calculated for C_22_H_19_N_3_O_4_; 390.1446; found 390.1448.

## 6. Refinement

Crystal data, data collection and structure refinement details are summarized in Table 2 ▸. Hydrogen atoms were placed at geometrical positions, except for the N—H hydrogen atom, the position of which was located in a difference-Fourier map and freely refined. The Flack parameter of −0.8 (9) indicates that the absolute structure cannot confidently be derived from the data based on Mo radiation.

## Supplementary Material

Crystal structure: contains datablock(s) I, global. DOI: 10.1107/S2056989023003948/wm5682sup1.cif

Structure factors: contains datablock(s) I. DOI: 10.1107/S2056989023003948/wm5682Isup2.hkl

Supporting information file. DOI: 10.1107/S2056989023003948/wm5682Isup3.cml

CCDC reference: 2232132

Additional supporting information:  crystallographic information; 3D view; checkCIF report

## Figures and Tables

**Table 1:** Hydrogen-bond geometry (Å, °)

*D*—H⋯*A*	*D*—H	H⋯*A*	*D*⋯*A*	*D*—H⋯*A*
C14—H14⋯N2^i^	0.95	2.68	3.524 (5)	148
C16—H16⋯O2^ii^	0.95	2.45	3.259 (4)	143
C21—H21⋯O4^iii^	0.95	2.59	3.532 (5)	171
N1—H1⋯O2^ii^	0.90 (4)	2.04 (4)	2.911 (4)	161 (3)

Symmetry codes: (i) 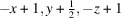 ; (ii) 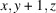 ; (iii) 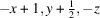 .

**Table 2:** Experimental details

Crystal data	
Chemical formula	C_22_H_19_N_3_O_4_
*M*_r_	389.40
Crystal system, space group	Monoclinic, *P*2_1_
Temperature (K)	173
*a*, *b*, *c* (Å)	8.9485 (8), 5.0612 (5), 20.949 (2)
β (°)	96.585 (7)
*V* (Å^3^)	942.54 (16)
*Z*	2
Radiation type	Mo *K*α
μ (mm^−1^)	0.10
Crystal size (mm)	0.30 × 0.28 × 0.03

Data collection	
Diffractometer	Rigaku R-AXIS RAPID
Absorption correction	Multi-scan (*ABSCOR*; Higashi, 1995 ▸)
*T*_min_, *T*_max_	0.749, 0.997
No. of measured, independent and observed [*I* > 2σ(*I*)] reflections	9086, 3799, 2635
*R*_int_	0.050
(sin θ/λ)_max_ (Å^−1^)	0.649

Refinement	
*R*[*F* ^2^ > 2σ(*F* ^2^)], *wR*(*F* ^2^), *S*	0.053, 0.126, 1.04
No. of reflections	3799
No. of parameters	266
No. of restraints	1
H-atom treatment	H atoms treated by a mixture of independent and constrained refinement
Δρ_max_, Δρ_min_ (e Å^−3^)	0.19, −0.16
Absolute structure	Unknown: Flack *x* determined using 741 quotients [(*I* ^+^)−(*I* ^−^)]/[(*I* ^+^)+(*I* ^−^)] (Parsons *et al.*, 2013 ▸)
Absolute structure parameter	−0.8 (9)

Computer programs: *RAPID-AUTO* (Rigaku, 2018 ▸), *SHELXT* (Sheldrick, 2015*a* ▸), *SHELXL* (Sheldrick, 2015*b* ▸), *DIAMOND* (Brandenburg, 1999 ▸) and *WinGX* (Farrugia, 2012 ▸).

## supplementary crystallographic information

### Crystal data

**Table:**

C_22_H_19_N_3_O_4_	*F*(000) = 408
*M**_r_* = 389.40	*D*_x_ = 1.372 Mg m^−^^3^
Monoclinic, *P*2_1_	Mo *K*α radiation, λ = 0.71075 Å
*a* = 8.9485 (8) Å	Cell parameters from 5984 reflections
*b* = 5.0612 (5) Å	θ = 2.3–27.5°
*c* = 20.949 (2) Å	µ = 0.10 mm^−^^1^
β = 96.585 (7)°	*T* = 173 K
*V* = 942.54 (16) Å^3^	Platel, colorless
*Z* = 2	0.30 × 0.28 × 0.03 mm

### Data collection

**Table:**

Rigaku R-AXIS RAPID diffractometer	2635 reflections with *I* > 2σ(*I*)
Detector resolution: 10.000 pixels mm^-1^	*R*_int_ = 0.050
ω scans	θ_max_ = 27.5°, θ_min_ = 2.0°
Absorption correction: multi-scan (*ABSCOR*; Higashi, 1995)	*h* = −11→11
*T*_min_ = 0.749, *T*_max_ = 0.997	*k* = −5→6
9086 measured reflections	*l* = −27→27
3799 independent reflections	

### Refinement

**Table:**

Refinement on *F*^2^	Hydrogen site location: mixed
Least-squares matrix: full	H atoms treated by a mixture of independent and constrained refinement
*R*[*F*^2^ > 2σ(*F*^2^)] = 0.053	*w* = 1/[σ^2^(*F*_o_^2^) + (0.0637*P*)^2^ + 0.0221*P*] where *P* = (*F*_o_^2^ + 2*F*_c_^2^)/3
*wR*(*F*^2^) = 0.126	(Δ/σ)_max_ = 0.001
*S* = 1.03	Δρ_max_ = 0.19 e Å^−^^3^
3799 reflections	Δρ_min_ = −0.16 e Å^−^^3^
266 parameters	Absolute structure: Flack *x* determined using 741 quotients [(*I*^+^)-(*I*^-^)]/[(*I*^+^)+(*I*^-^)] (Parsons *et al.*, 2013)
1 restraint	Absolute structure parameter: −0.8 (9)

### Special details

**Table:**

Geometry. All esds (except the esd in the dihedral angle between two l.s. planes) are estimated using the full covariance matrix. The cell esds are taken into account individually in the estimation of esds in distances, angles and torsion angles; correlations between esds in cell parameters are only used when they are defined by crystal symmetry. An approximate (isotropic) treatment of cell esds is used for estimating esds involving l.s. planes.

### Fractional atomic coordinates and isotropic or equivalent isotropic displacement parameters (Å^2^)

**Table:**

	*x*	*y*	*z*	*U*_iso_*/*U*_eq_	
O1	0.1477 (3)	0.5424 (5)	0.66816 (11)	0.0476 (7)	
O2	0.3072 (3)	0.1188 (5)	0.39878 (10)	0.0407 (6)	
O3	0.7195 (4)	0.3955 (11)	0.04333 (15)	0.0967 (14)	
O4	0.5966 (4)	0.7477 (8)	0.00909 (14)	0.0786 (10)	
N1	0.3611 (4)	0.5542 (5)	0.38830 (13)	0.0369 (7)	
H1	0.354 (4)	0.724 (8)	0.4011 (17)	0.044*	
N2	0.4173 (3)	0.5102 (5)	0.33080 (12)	0.0359 (7)	
N3	0.6379 (4)	0.5860 (10)	0.05105 (16)	0.0632 (11)	
C1	−0.1011 (5)	0.8823 (12)	0.93031 (19)	0.0716 (14)	
H1A	−0.136189	1.063016	0.920871	0.086*	
H1B	−0.010078	0.886972	0.961152	0.086*	
H1C	−0.179652	0.782238	0.948641	0.086*	
C2	−0.0660 (4)	0.7506 (9)	0.86904 (16)	0.0472 (10)	
C3	0.0290 (5)	0.5425 (10)	0.86983 (18)	0.0648 (13)	
H3	0.074131	0.476727	0.909949	0.078*	
C4	0.0622 (5)	0.4232 (10)	0.81397 (18)	0.0661 (13)	
H4	0.130000	0.278228	0.816291	0.079*	
C5	−0.0019 (4)	0.5115 (8)	0.75470 (16)	0.0401 (8)	
C6	−0.0974 (5)	0.7189 (9)	0.75353 (18)	0.0554 (11)	
H6	−0.143298	0.783136	0.713380	0.067*	
C7	−0.1295 (5)	0.8387 (11)	0.80936 (19)	0.0655 (13)	
H7	−0.196585	0.984825	0.806939	0.079*	
C8	0.0349 (4)	0.3846 (8)	0.69368 (16)	0.0462 (9)	
H8A	0.072933	0.203070	0.702463	0.055*	
H8B	−0.056490	0.374539	0.662284	0.055*	
C9	0.1823 (4)	0.4840 (7)	0.60809 (15)	0.0360 (8)	
C10	0.1222 (4)	0.2735 (7)	0.57052 (15)	0.0387 (9)	
H10	0.052049	0.156850	0.586470	0.046*	
C11	0.1652 (4)	0.2362 (7)	0.51033 (15)	0.0382 (8)	
H11	0.123802	0.092481	0.485007	0.046*	
C12	0.2679 (4)	0.4034 (6)	0.48530 (15)	0.0311 (8)	
C13	0.3289 (4)	0.6103 (7)	0.52395 (15)	0.0388 (8)	
H13	0.399403	0.727021	0.508241	0.047*	
C14	0.2876 (4)	0.6465 (7)	0.58475 (16)	0.0419 (9)	
H14	0.332136	0.785035	0.610979	0.050*	
C15	0.3126 (4)	0.3454 (7)	0.42105 (15)	0.0318 (8)	
C16	0.4317 (4)	0.7143 (7)	0.29601 (16)	0.0410 (8)	
H16	0.404496	0.884332	0.310034	0.049*	
C17	0.4910 (4)	0.6807 (7)	0.23383 (16)	0.0405 (9)	
C18	0.5876 (5)	0.4754 (8)	0.22322 (18)	0.0463 (9)	
H18	0.620600	0.356662	0.257043	0.056*	
C19	0.6358 (5)	0.4437 (9)	0.16319 (18)	0.0509 (10)	
H19	0.702781	0.304857	0.155381	0.061*	
C20	0.5843 (4)	0.6185 (9)	0.11485 (17)	0.0491 (10)	
C21	0.4894 (5)	0.8217 (8)	0.12357 (17)	0.0545 (11)	
H21	0.455193	0.937239	0.089234	0.065*	
C22	0.4445 (5)	0.8546 (8)	0.18362 (17)	0.0526 (10)	
H22	0.380496	0.998273	0.191162	0.063*	

### Atomic displacement parameters (Å^2^)

**Table:**

	*U*^11^	*U*^22^	*U*^33^	*U*^12^	*U*^13^	*U*^23^
O1	0.0515 (15)	0.0606 (18)	0.0328 (13)	−0.0128 (14)	0.0139 (11)	−0.0079 (12)
O2	0.0605 (16)	0.0307 (12)	0.0321 (12)	0.0011 (13)	0.0101 (11)	−0.0015 (11)
O3	0.076 (2)	0.168 (4)	0.0489 (19)	0.040 (3)	0.0199 (16)	−0.007 (2)
O4	0.113 (3)	0.086 (2)	0.0419 (17)	−0.015 (2)	0.0324 (17)	0.0032 (17)
N1	0.0571 (18)	0.0288 (16)	0.0259 (14)	0.0000 (15)	0.0105 (12)	−0.0017 (12)
N2	0.0492 (17)	0.0322 (15)	0.0274 (15)	0.0007 (14)	0.0087 (12)	−0.0020 (12)
N3	0.059 (2)	0.094 (3)	0.038 (2)	−0.011 (2)	0.0142 (17)	−0.008 (2)
C1	0.065 (3)	0.110 (4)	0.043 (2)	−0.008 (3)	0.023 (2)	−0.018 (2)
C2	0.0412 (19)	0.068 (3)	0.035 (2)	−0.014 (2)	0.0156 (15)	−0.0043 (19)
C3	0.092 (3)	0.069 (3)	0.031 (2)	0.011 (3)	−0.002 (2)	0.0080 (19)
C4	0.083 (3)	0.070 (3)	0.044 (2)	0.034 (3)	−0.001 (2)	0.002 (2)
C5	0.0385 (19)	0.049 (2)	0.0337 (18)	−0.0054 (19)	0.0090 (15)	−0.0003 (16)
C6	0.064 (2)	0.065 (3)	0.035 (2)	0.015 (2)	−0.0024 (18)	0.0006 (19)
C7	0.055 (2)	0.094 (4)	0.046 (2)	0.025 (3)	0.0002 (19)	−0.015 (2)
C8	0.048 (2)	0.056 (2)	0.037 (2)	−0.008 (2)	0.0126 (16)	−0.0022 (18)
C9	0.0385 (19)	0.042 (2)	0.0282 (17)	0.0001 (17)	0.0049 (14)	−0.0003 (15)
C10	0.0410 (19)	0.037 (2)	0.0396 (19)	−0.0052 (17)	0.0121 (16)	−0.0015 (16)
C11	0.0473 (19)	0.0326 (17)	0.0349 (19)	−0.0044 (17)	0.0051 (15)	−0.0069 (15)
C12	0.0395 (19)	0.0279 (17)	0.0259 (16)	0.0045 (16)	0.0035 (14)	−0.0016 (13)
C13	0.0467 (19)	0.039 (2)	0.0311 (17)	−0.0066 (18)	0.0073 (15)	−0.0001 (16)
C14	0.049 (2)	0.043 (2)	0.0333 (19)	−0.0072 (19)	0.0019 (15)	−0.0074 (16)
C15	0.0373 (19)	0.0297 (18)	0.0276 (16)	0.0041 (16)	0.0006 (14)	−0.0006 (14)
C16	0.061 (2)	0.0318 (18)	0.0320 (18)	−0.0014 (18)	0.0108 (16)	−0.0054 (15)
C17	0.056 (2)	0.036 (2)	0.0312 (18)	−0.0137 (18)	0.0114 (16)	−0.0051 (15)
C18	0.054 (2)	0.048 (2)	0.038 (2)	−0.008 (2)	0.0104 (17)	−0.0014 (17)
C19	0.051 (2)	0.057 (2)	0.047 (2)	−0.009 (2)	0.0170 (18)	−0.010 (2)
C20	0.051 (2)	0.068 (3)	0.0306 (19)	−0.019 (2)	0.0144 (16)	−0.007 (2)
C21	0.079 (3)	0.054 (3)	0.032 (2)	−0.018 (2)	0.0133 (19)	0.0022 (18)
C22	0.082 (3)	0.040 (2)	0.039 (2)	−0.006 (2)	0.021 (2)	0.0025 (17)

### Geometric parameters (Å, º)

**Table:**

O1—C9	1.363 (4)	C8—H8A	0.9900
O1—C8	1.437 (4)	C8—H8B	0.9900
O2—C15	1.237 (4)	C9—C14	1.382 (5)
O3—N3	1.231 (6)	C9—C10	1.395 (5)
O4—N3	1.227 (5)	C10—C11	1.373 (4)
N1—C15	1.358 (4)	C10—H10	0.9500
N1—N2	1.376 (4)	C11—C12	1.395 (5)
N1—H1	0.90 (4)	C11—H11	0.9500
N2—C16	1.279 (4)	C12—C13	1.396 (5)
N3—C20	1.480 (5)	C12—C15	1.477 (4)
C1—C2	1.511 (5)	C13—C14	1.379 (5)
C1—H1A	0.9800	C13—H13	0.9500
C1—H1B	0.9800	C14—H14	0.9500
C1—H1C	0.9800	C16—C17	1.472 (5)
C2—C3	1.353 (6)	C16—H16	0.9500
C2—C7	1.386 (5)	C17—C18	1.385 (5)
C3—C4	1.379 (6)	C17—C22	1.398 (5)
C3—H3	0.9500	C18—C19	1.385 (5)
C4—C5	1.381 (5)	C18—H18	0.9500
C4—H4	0.9500	C19—C20	1.383 (6)
C5—C6	1.352 (6)	C19—H19	0.9500
C5—C8	1.500 (5)	C20—C21	1.360 (6)
C6—C7	1.377 (5)	C21—C22	1.374 (5)
C6—H6	0.9500	C21—H21	0.9500
C7—H7	0.9500	C22—H22	0.9500
			
C9—O1—C8	117.8 (3)	C14—C9—C10	119.3 (3)
C15—N1—N2	119.1 (3)	C11—C10—C9	119.5 (3)
C15—N1—H1	124 (2)	C11—C10—H10	120.2
N2—N1—H1	117 (2)	C9—C10—H10	120.2
C16—N2—N1	116.0 (3)	C10—C11—C12	121.9 (3)
O4—N3—O3	124.3 (4)	C10—C11—H11	119.1
O4—N3—C20	118.1 (4)	C12—C11—H11	119.1
O3—N3—C20	117.7 (4)	C11—C12—C13	117.9 (3)
C2—C1—H1A	109.5	C11—C12—C15	118.8 (3)
C2—C1—H1B	109.5	C13—C12—C15	123.3 (3)
H1A—C1—H1B	109.5	C14—C13—C12	120.4 (3)
C2—C1—H1C	109.5	C14—C13—H13	119.8
H1A—C1—H1C	109.5	C12—C13—H13	119.8
H1B—C1—H1C	109.5	C13—C14—C9	120.9 (3)
C3—C2—C7	117.0 (4)	C13—C14—H14	119.5
C3—C2—C1	121.6 (4)	C9—C14—H14	119.5
C7—C2—C1	121.4 (4)	O2—C15—N1	122.1 (3)
C2—C3—C4	121.8 (4)	O2—C15—C12	121.7 (3)
C2—C3—H3	119.1	N1—C15—C12	116.2 (3)
C4—C3—H3	119.1	N2—C16—C17	118.7 (3)
C3—C4—C5	120.9 (4)	N2—C16—H16	120.6
C3—C4—H4	119.5	C17—C16—H16	120.6
C5—C4—H4	119.5	C18—C17—C22	119.3 (3)
C6—C5—C4	117.6 (4)	C18—C17—C16	121.6 (3)
C6—C5—C8	121.1 (3)	C22—C17—C16	119.1 (3)
C4—C5—C8	121.2 (4)	C19—C18—C17	119.8 (4)
C5—C6—C7	121.3 (4)	C19—C18—H18	120.1
C5—C6—H6	119.3	C17—C18—H18	120.1
C7—C6—H6	119.3	C20—C19—C18	118.6 (4)
C6—C7—C2	121.4 (4)	C20—C19—H19	120.7
C6—C7—H7	119.3	C18—C19—H19	120.7
C2—C7—H7	119.3	C21—C20—C19	123.1 (3)
O1—C8—C5	108.1 (3)	C21—C20—N3	118.5 (4)
O1—C8—H8A	110.1	C19—C20—N3	118.4 (4)
C5—C8—H8A	110.1	C20—C21—C22	117.9 (4)
O1—C8—H8B	110.1	C20—C21—H21	121.1
C5—C8—H8B	110.1	C22—C21—H21	121.1
H8A—C8—H8B	108.4	C21—C22—C17	121.3 (4)
O1—C9—C14	115.7 (3)	C21—C22—H22	119.3
O1—C9—C10	125.0 (3)	C17—C22—H22	119.3
			
C15—N1—N2—C16	166.0 (3)	C10—C9—C14—C13	2.9 (5)
C7—C2—C3—C4	0.3 (7)	N2—N1—C15—O2	−5.3 (5)
C1—C2—C3—C4	−179.5 (5)	N2—N1—C15—C12	174.1 (3)
C2—C3—C4—C5	−0.5 (8)	C11—C12—C15—O2	−25.7 (5)
C3—C4—C5—C6	0.2 (7)	C13—C12—C15—O2	151.0 (3)
C3—C4—C5—C8	179.3 (4)	C11—C12—C15—N1	154.8 (3)
C4—C5—C6—C7	0.2 (6)	C13—C12—C15—N1	−28.5 (5)
C8—C5—C6—C7	−178.9 (4)	N1—N2—C16—C17	−179.9 (3)
C5—C6—C7—C2	−0.4 (7)	N2—C16—C17—C18	−27.8 (5)
C3—C2—C7—C6	0.2 (7)	N2—C16—C17—C22	149.9 (4)
C1—C2—C7—C6	179.9 (4)	C22—C17—C18—C19	−0.4 (5)
C9—O1—C8—C5	−170.9 (3)	C16—C17—C18—C19	177.2 (4)
C6—C5—C8—O1	80.9 (4)	C17—C18—C19—C20	−0.6 (5)
C4—C5—C8—O1	−98.2 (5)	C18—C19—C20—C21	0.5 (6)
C8—O1—C9—C14	177.9 (3)	C18—C19—C20—N3	179.1 (4)
C8—O1—C9—C10	−3.5 (5)	O4—N3—C20—C21	1.5 (5)
O1—C9—C10—C11	179.5 (3)	O3—N3—C20—C21	−177.5 (4)
C14—C9—C10—C11	−2.0 (5)	O4—N3—C20—C19	−177.2 (4)
C9—C10—C11—C12	0.0 (5)	O3—N3—C20—C19	3.9 (5)
C10—C11—C12—C13	1.1 (5)	C19—C20—C21—C22	0.7 (6)
C10—C11—C12—C15	177.9 (3)	N3—C20—C21—C22	−177.9 (3)
C11—C12—C13—C14	−0.2 (5)	C20—C21—C22—C17	−1.8 (6)
C15—C12—C13—C14	−176.9 (3)	C18—C17—C22—C21	1.7 (6)
C12—C13—C14—C9	−1.8 (5)	C16—C17—C22—C21	−176.0 (3)
O1—C9—C14—C13	−178.5 (3)		

### Hydrogen-bond geometry (Å, º)

**Table:**

*D*—H···*A*	*D*—H	H···*A*	*D*···*A*	*D*—H···*A*
C14—H14···N2^i^	0.95	2.68	3.524 (5)	148
C16—H16···O2^ii^	0.95	2.45	3.259 (4)	143
C21—H21···O4^iii^	0.95	2.59	3.532 (5)	171
N1—H1···O2^ii^	0.90 (4)	2.04 (4)	2.911 (4)	161 (3)

Symmetry codes: (i) −*x*+1, *y*+1/2, −*z*+1; (ii) *x*, *y*+1, *z*; (iii) −*x*+1, *y*+1/2, −*z*.

## References

[bb1] Allen, F. H., Kennard, O., Watson, D. G., Brammer, L., Orpen, A. G. & Taylor, R. (1987). *J. Chem. Soc. Perkin Trans. 2*, pp. S1–S19.

[bb2] Ban, H.-Y. & Li, C.-M. (2009). *Acta Cryst.* E**65**, o3272. 10.1107/S160053680905079X PMC2972042 21578966

[bb3] Banna, M. H. A., Howlader, M. B. H., Miyatake, R., Sheikh, M. C., Ansary, M. R. H. & Zangrando, E. (2023). *Acta Cryst.* E**79**, 207–211. 10.1107/S2056989023001354 PMC9993917 36909983

[bb4] Banna, M. H. A., Howlader, M. B. H., Miyatake, R., Sheikh, M. C. & Zangrando, E. (2022). *Acta Cryst.* E**78**, 1081–1083. 10.1107/S2056989022009392 PMC9535816 36250110

[bb5] Brandenburg, K. (1999). *DIAMOND.* Crystal Impact GbR, Bonn, Germany.

[bb6] Chantrapromma, S., Prachumrat, P., Ruanwas, P., Boonnak, N. & Kassim, M. B. (2016). *Acta Cryst.* E**72**, 1339–1342. 10.1107/S2056989016013268 PMC5120720 27920930

[bb7] Farrugia, L. J. (2012). *J. Appl. Cryst.* **45**, 849–854.

[bb8] Higashi, T. (1995). *ABSCOR*. Rigaku Corporation, Tokyo, Japan.

[bb9] Horkaew, J., Chantrapromma, S. & Fun, H.-K. (2011). *Acta Cryst.* E**67**, o2985. 10.1107/S1600536811041535 PMC3247387 22220005

[bb10] Parsons, S., Flack, H. D. & Wagner, T. (2013). *Acta Cryst.* B**69**, 249–259. 10.1107/S2052519213010014 PMC3661305 23719469

[bb11] Rigaku (2018). *RAPID-AUTO*. Rigaku Corporation, Tokyo, Japan.

[bb12] Sheldrick, G. M. (2015*a*). *Acta Cryst.* A**71**, 3–8.

[bb13] Sheldrick, G. M. (2015*b*). *Acta Cryst.* C**71**, 3–8.

[bb14] Taha, M., Ismail, N. H., Jamil, W., Yousuf, S., Jaafar, F. M., Ali, M. I., Kashif, S. M. & Hussain, E. (2013). *Molecules*, **18**, 10912–10929. 10.3390/molecules180910912 PMC6269687 24013406

[bb15] Verma, G., Marella, A., Shaquiquzzaman, M., Akhtar, M., Ali, M. R. & Alam, M. M. (2014). *J. Pharm. Bioallied. Sci.* **6**, 69–80. 10.4103/0975-7406.129170 PMC3983749 24741273

[bb16] Zong, Q.-S. & Wu, J.-Y. (2013). *J. Struct. Chem.* **54**, 1151–1156.

[bb17] Zülfikaroğlu, A., Yüksektepe Ataol, Ç., Çelikoğlu, E., Çelikoğlu, U. & İdil, Ö. (2020). *J. Mol. Struct.* **1199**, 127012.

